# The Impact of Physical Exercise on Male Fertility Through Its Association with Various Processes and Aspects of Human Biology

**DOI:** 10.3390/jcm14103442

**Published:** 2025-05-14

**Authors:** Adrianna Zańko, Michał Pawłowski, Robert Milewski

**Affiliations:** Department of Biostatistics and Medical Informatics, Medical University of Białystok, 15-295 Białystok, Poland; adrianna.zanko@umb.edu.pl (A.Z.); michal.pawlowski@umb.edu.pl (M.P.)

**Keywords:** male fertility, physical activity, infertility, lifestyle

## Abstract

**Background/Objective:** Infertility affects approximately 10–15% couples in industrialized countries. It has numerous causes, including genetic and environmental factors, lifestyle choices, and physiological disorders. The increasing prevalence of infertility underlines the importance of research into interventions to improve reproductive health, with a strong focus on physical activity. Infertility research was traditionally mainly directed toward female health. Although the male factor is being increasingly accepted as being equally important, this area remains under-researched. The current review focuses on the impact of physical activity on male fertility through its effects on immune function, the cardiovascular system, hormonal balance, metabolism, and physical interaction with the male reproductive system. **Materials and Methods:** A comprehensive literature review of studies addressing the effects of physical activity on male fertility was conducted using PubMed/Medline, Scopus, and the Web of Science. Mostly recent studies were included, with a small number of older ones included in cases when their content remains relevant. The review focused on articles studying the processes involved and associations between physical activity and male fertility through immune and cardiovascular effects, endocrine modulation, the influence on obesity and insulin metabolism, and the physical impact on the body. **Results:** The findings revealed the existence of a fairly strong consensus that moderate physical activity enhances semen quality, hormonal balance, and metabolic health, positively influencing male fertility. Physical activity reduces inflammation and oxidative stress, enhances cardiovascular functioning, and contributes to oxygen and nutrient supply to the reproductive organs. On the contrary, strenuous training can adversely affect fertility, mostly through hormonal disruption and oxidative stress. It can also have various indirect effects on fertility through sports-related behavior and incidents, such as wearing tight-fitting clothes or overheating. **Conclusions:** Physical activity can affect male fertility in numerous ways, positively influencing reproductive health when performed at a moderate intensity. Understanding the balance between beneficial and excessive exercise as well as the impact of incidental factors related to performing sports regularly are, thus, extremely important in optimizing lifestyle-oriented interventions aimed at male fertility improvement.

## 1. Introduction

Infertility affects around 10–15 percent of couples in developed countries, according to the latest epidemiological data. According to the most recent definition, infertility is the inability to conceive a child after one year or more of regular, unprotected sexual intercourse during the fertile phase of the menstrual cycle. In the case of males, the condition is defined by the presence of suboptimal sperm parameters in the male partner of a couple of reproductive age, except in cases of idiopathic infertility, which is largely linked to lifestyle factors and affects more than 42 million people worldwide [1,2,3]. It must be emphasized that the strategy to help couples facing infertility issues often focuses solely on women [4,5]. This traditional approach to fertility treatment leads to insufficient attention being given to the male factor, which results in men often lacking both the necessary knowledge [6] and support to take proactive steps to improve fertility. For this reason, both research studies and clinical guidelines are urgently needed to improve this area of human fertility.

The rate of male infertility is constantly increasing, with global age-standardized prevalence rates of infertility per 100,000 population having increased by 19% between 1990 and 2019. In addition, infertility rates are income dependent, with male infertility rates in high–middle and middle Socio-demographic Index (SDI) regions exceeding the global average. As far as particular regions are concerned, the highest rates of change were observed for Central Latin America (62.8%) and Western Sub-Saharan Africa (57.5%). Increasing infertility rates were also observed in high-income locations such as High-income North America (41.6%) and Western Europe (34.8%). Interestingly, however, High-income Asia Pacific experienced a slight decrease in infertility rates (−0.6%), making it one of only three regions where the prevalence rates of infertility declined in the studied period. The other regions showing a decrease were Central Sub-Saharan Africa (1.2%) and Oceania (−0.3%) [5].

Numerous causes of male infertility have been identified. Epidemiological research suggests that environmental pollution, i.e., organic, inorganic, and air pollutants; workplace exposures, including high temperatures, organic solvents, and pesticides; and unhealthy lifestyle choices, such as poor diet, lack of sleep, smoking, alcohol use, and inadequate exercise, play significant roles as non-genetic causes of male infertility [7]. For diagnostic purposes, the causes of infertility are categorized as follows: pre-testicular, testicular, post-testicular [8,9], and sexual disorders. Pre-testicular causes include environmental hazards, unhealthy lifestyle choices, and disturbances in the secretion or action of pituitary gonadotropins, steroid hormones, and thyroid hormones. The testicular causes are either congenital or acquired disorders of the testicular structure, orchitis, mechanical injuries to the testicles, torsion of the testicle, prolonged ischemia of the gonad, and cryptorchidism. Post-testicular causes include mechanical injuries to the epididymis, vas deferens, or urethra, vasectomies, obstructions of semen transport pathways, hypoplasia of the epididymis or vas deferens, and inflammation of the accessory glands. Sexual disorders include structural abnormalities of the penis that prevent sexual intercourse, as well as erectile dysfunction and anejaculation [8]. Leslie et al. [9] use a similar classification to report the following specific percentages for the various causes of male infertility: primary testicular defects (65–80%), idiopathic infertility (10–20%), sperm transport disorders (5%), and endocrine disorders (2–5%).

The basic test used for the assessment of male infertility is semen analysis, performed after a period of sexual abstinence lasting from 2 to 7 days and analyzed using computer-assisted semen analysis (CASA) [8,10]. The latest standards, included in the sixth edition of the WHO Manual for the Laboratory Examination and Processing of Human Semen [8], indicate that semen volume should be at least 1.4 mL, while the optimal total sperm number should be 39 × 10^6^ per ejaculate. The optimal total motility should be at least 40%, with progressive motility at 32% or higher. Morphology, on the other hand, should exceed 4% normal forms [8,11]. A patient with abnormal semen parameters should have the test repeated after a period of at least one month. If the second test confirms the abnormalities, more detailed diagnostic evaluation should be conducted [12].

Physical activity is the foundation of a healthy lifestyle. Due to the significant impact of insufficient physical activity on disease occurrence, it is estimated that over 5 million deaths per year could be prevented by incorporating an appropriate level of exercise [13]. In addition, physical activity has an impact on male fertility, which has been a topic of research for some time. Although some studies suggest no association between physical activity and male fertility [14,15], and others report mixed results [16], the vast majority of research indicates a positive impact of physical activity on male fertility [17,18,19,20,21,22,23,24,25]. In particular, the literature data point to the possibility of an impact of physical activity on semen parameters. For example, Donato et al. [26] conducted a prospective cohort study whose results indicate that the highest total motility and normal morphology values were associated with medium-level physical activity, while the lowest values were seen in the case of both reduced and increased activity. Other studies confirm that physical exercise is associated with improved semen parameters, such as sperm concentration, total sperm motility, total sperm count, and normal morphology [18,27]. It has also been suggested that various forms of intense or excessive physical training can have harmful effects on both the overall and reproductive health [2,23,26,28,29]. Interestingly, however, certain studies report no negative influence of excessive exercise [18,27].

It has been increasingly widely acknowledged in the scientific literature that lifestyle interventions improve a wide range of aspects connected with male fertility [28]. One key mechanism involves osteocalcin, a hormone released by bones during physical activity. Osteocalcin plays a role in regulating glucose and fatty acid metabolisms, improving insulin sensitivity, and promoting the synthesis of testosterone by affecting its production in Leydig cells, which is vital for many aspects of testicular function [30,31,32]. The results of a study conducted by Mera et al. [33] demonstrated that osteocalcin is found circulating in the bloodstream after a single running session on the treadmill. Physical activity has also been found to be associated with improved pregnancy outcomes. A systematic review and meta-analysis based on 336 studies identified combined aerobic and resistance training as the most beneficial approach as far as pregnancy outcomes are concerned, simultaneously emphasizing the fact that physical activity in general leads to improved male fertility, compared to no intervention [17]. Additionally, research has linked a sedentary lifestyle to a higher risk of erectile dysfunction, further underscoring the importance of regular exercise for reproductive health [34,35].

Apart from studying the impact of physical activity on male reproductive health in general and semen parameters in particular, scientific papers also focus on the assessment of the impact of specific sports disciplines [19,20,36], a particular type of physical activity [21,22,23], or the intensity of physical activity on male fertility [24,25,36]. Understanding the impact of specific processes occurring in a man’s body during physical activity, which indirectly or directly affect his body and fertility, seems to be an interesting and valuable research direction, with interactions between the endocrine and immune systems and energy metabolism often indicated as the key physiological processes involved in male reproduction [37].

The aim of this study was to review the available scientific literature concerning the relationship between physical exercise and male fertility, focusing on the influence of physical activity on various aspects of male reproductive health. Specifically, the study aimed to analyze the available data on the associations between physical activity and male fertility through its effects on key physiological processes, such as immune function, cardiovascular health, hormonal regulation, insulin metabolism, and the physical action on the male body. It must be noted that studying the influence of physical exercise on male reproductive potential is challenging due to the large number of confounders, particularly the following:

− Diet (e.g., supplementation with antioxidants, micronutrients, and vitamins);
− Sleep (e.g., low-quality sleep or sleep deprivation);
− Stress;
− Environmental pollution (e.g., exposure to heavy metals and other pollutants);
− Substance use (e.g., alcohol or drugs);
− Type of work (e.g., sedentary occupations);
− Overheating (e.g., using Jacuzzi or sauna or wearing tight-fitting clothes);
− Exposure to radiation (e.g., carrying mobile phones in trouser pockets or undergoing radiotherapy) [38].

It must be noted that—as mentioned earlier—not only are most of these confounders identified as the main lifestyle-related causes of male infertility [7], but they also tend to co-exist in the same individuals, making establishing causal relationships particularly difficult. However, despite the problems inherent in dealing with large numbers of interlinked confounders, in light of the available literature data, certain conclusions seem particularly convincing.

## 2. Materials and Methods

This study was conducted as a literature review of publications sourced from databases such as PubMed/Medline, EMBASE (Elsevier), Scopus, the Web of Science, and Google Scholar. The majority of the included articles were published within the last two decades. The following queries were used in the search for publications:

− (Physical activity or interval training or resistance training or endurance training) and (male fertility or semen quality);
− (Physical activity or interval training or resistance training or endurance training) and (immune system or cardiovascular system or obesity and insulin resistance or endocrine system or physical trauma or overheating of the body or extreme sports);
− (Male fertility or semen quality) and (immune system or cardiovascular system or obesity and insulin resistance or endocrine system or physical trauma or overheating of the body or extreme sports).

The review aimed to analyze the current scientific understanding of the underlying processes and the various associations between physical exercise and male fertility. The review was divided into five sections, each devoted to connections between physical activity and male reproductive health through its influence on the following areas:

1. The immune system;
2. Endocrine action;
3. The circulatory system;
4. Actions related to insulin resistance, diabetes, and obesity;
5. Physical action on the male body.

While the authors prioritized recent research, older studies were also incorporated if they were considered relevant, particularly systematic reviews or meta-analyses. In particular, the authors referred to older publications in cases when no recent articles were found on a given issue. Although no strict exclusion criteria were applied, each article identified through the queries was assessed for its content to ensure it was relevant to the subject matter of the study.

## 3. Discussion

Table 1 presents a summary of the main findings of the review concerning the impact of various aspects of the human biology on male fertility.

In terms of the differentiation between low, moderate, and high amounts of physical activity, some of the analyzed articles defined cutoff thresholds for different training intensities in considerable detail, while others did not provide such specifics and used a more intuitive classification. Additionally, some articles used a different number of categories, e.g., only distinguishing between activity and inactivity or dividing physical activity into more than three levels, establishing several degrees of high or low intensity. To synthesize the findings, the categorizations suggested by the authors of the individual studies were followed. As the lack of consensus regarding the cutoff values is problematic in itself, a unified approach for future studies would be beneficial. Hence, the authors of this study have identified the classification proposed by Donato et al. [26], based on the IPAQ5 questionnaire, as the most reliable. This approach involves summing the results of walking, moderate, and vigorous physical activities and then categorizing the participants into three groups: low (<600 MET), moderate (600–2999 MET), and high (≥3000 MET) physical activity. Adopting such a standardized and objectified approach could streamline the methodology involved in research into the role of physical exercise in male fertility.

### 3.1. Impact of Physical Activity on Fertility Through Its Effect on the Immune System

Due to their complexity, male reproductive processes are closely linked to various biological activities in the human body, including those involving the immune system [39]. Most importantly, immune tolerance mechanisms prevent the body from attacking germ cells during spermatogenesis. For this reason, although new, cell-specific antigens are introduced during spermatogenesis, fertility is not compromised because the immune system does not initiate a response to germ cells. This is due to several factors that contribute to this process [40,41], including the presence of biological components that create a protective microenvironment supporting the development and maturation of germ cells, such as the blood–testis barrier (BTB), immune cells with immunosuppressive activity, and somatic cells of the testes [37,40,42]. It is now known that this environment, regulated by various structural components and local immunoregulatory factors, is designed in a way that allows successful reproduction [43].

However, despite its protective activity, the immune system also faces challenges in the context of fertility. One such challenge is that metabolic imbalance, in spite of the presence of the blood–testis barrier, can lead to testicular dysfunction affecting spermatogenesis, potentially resulting in apoptosis of spermatogenic cells [18] and hypogonadism. Metabolic processes in the testes generate energy and biosynthetic precursors that regulate germ cell development and modulate testicular immunity and inflammation. Metabolism of immune cells, thus, plays a vital role in both inflammatory and anti-inflammatory responses, which are believed to influence spermatogenesis in the testes [37].

One of the immune mechanisms connected with impaired spermatogenesis involves adipose tissue. Metabolic disorders associated with adipose tissue impair spermatogenesis by increasing the conversion of testosterone to 17β-estradiol, inducing a feedback mechanism on the hypothalamic–pituitary–testicular (HPT) axis. In this manner, the secretion of sex hormones such as LH and FSH is reduced [37,44]. The suppression of the HPT axis by adipose tissue may result from dysregulated leptin signaling and the production of pro-inflammatory cytokines [45].

For the reasons outlined above, the relationship between physical exercise, the immune system, and oxidative processes should be regarded as complex and remains the subject of ongoing research. Physical activity has been shown to be associated with improved semen quality and spermatogenesis in cases of lifestyle-induced infertility caused by increased testicular antioxidant defense, reducing pro-inflammatory cytokine levels and enhancing steroidogenesis [2,46]. Pro-inflammatory cytokines are known to reduce sperm quality, causing DNA damage, among other disruptions [47]. Moreover, two combinations of pro-inflammatory cytokines, i.e., IL-6 + IL-8 and IL-12 + IL-18, have been found to enhance sperm membrane lipid peroxidation caused, in most cases, by leukocytes [48]. Some cytokines, e.g., IL-1β, TNF-α, or IL-18, are also known to participate in the regulation of apoptotic processes via the induction of the Fas/Fas ligand (FasL) system [47,49].

Engaging in an exercise session causes an increased release of the total number of white blood cells, granulocyte-associated proteins, and various plasma cytokines, including interleukin-6 (IL-6), IL-8, IL-10, IL-18, interleukin-1 receptor antagonist (IL-1ra), granulocyte colony-stimulating factor, and monocyte chemoattractant protein [50,51,52]. Although the levels of the aforementioned inflammatory biomarkers consistently increase during intense exercise, physically fit individuals tend to exhibit lower resting levels compared to less fit persons. This is confirmed in the findings of a study performed by Nieman and Wentz [53], which showed that the average resting values of C-reactive protein (CRP) and interleukin-6 (IL-6) for obese groups and endurance athletes differed significantly, showing a 4.4-fold difference, while the IL-6 values exhibited a 1.3-fold difference, with higher levels observed in the case of obese subjects. In this context, the increased release of inflammatory factors during exercise can be seen as conditioning for the immune system to function more efficiently in the event of inflammation.

On the other hand, very intense physical exercise appears to cause oxidative tissue damage and trigger an immediate immune response [54]. The mechanisms involved include the stimulation of oxygen consumption and the formation of free radicals. An imbalance between antioxidant activity and the production of reactive oxygen species (ROS) leads to oxidative stress, which is associated with increased levels of inflammatory markers in the body [55]. Studies on rats have confirmed that both high-intensity running and intense swimming-based training can reduce enzymatic and non-enzymatic antioxidant activity in the testes, increase lipid peroxidation, and elevate ROS production [56,57].

Since the presence of inflammation is a known factor that disrupts male fertility, introducing a carefully crafted physical activity regimen may be beneficial for several reasons. For instance, thirty minutes after a high-intensity exercise session, the total antioxidant status in serum increases significantly, along with the ability to neutralize free radicals [58]. It has also been shown that regular physical activity has an anti-inflammatory effect, manifested in better regulation of inflammatory signaling pathways, the release of muscle myokines that stimulate the production of interleukin-1 and interleukin-10, reduced adipose tissue producing pro-inflammatory cytokines, improved oxygen utilization, enhanced innate immune function, and better oxylipin balance [59,60,61,62,63]. It has also been suggested that supplementation with the antioxidant L-carnitine in exercising men, at a dose as low as 2 g/day, has a positive impact on fertility by improving sperm parameters, regulating hormone levels, and reducing ROS levels [64]. Hence, the available studies clearly point to a consensus that due to the fact that physical exercise may be linked to better adaptation of genes for controlling inflammation and tissue repair, regular physical activity for reducing inflammation could have a protective effect on male fertility.

### 3.2. Impact of Physical Activity on Fertility Through Endocrine Action

The endocrine system supports the body’s homeostasis and adapts to external stimuli. Hormonal signaling plays various roles in both anabolism (tissue growth, repair, and regeneration) and catabolism (metabolic regulation and tissue breakdown). During training, hormonal mechanisms are part of a complex, integrated signaling system that indirectly modulates metabolic and cellular processes in skeletal muscles, the nervous system, and connective tissue. However, as hormones do not act in isolation, every hormonal or adaptive response must be considered in the context of the entire endocrine system and its interplay with other physiological systems [65].

From the physiological perspective, male reproduction results from the collaboration of several hormones responsible for spermatogenesis and sexual function. To produce these hormones, the hypothalamus, the pituitary gland, and the testes work together, which is why they are often treated as a single entity: the hypothalamic–pituitary–gonadal (HPG) axis. When a male reaches sexual maturity, gonadotropin-releasing hormone (GnRH) is released from hypothalamic neurons in pulses, stimulating the pituitary gland to produce two other hormones, namely luteinizing hormone (LH) and follicle-stimulating hormone (FSH), which then stimulate spermatogenesis and testosterone production in the testes [29,66]. Approximately 44–60% of the testosterone transported in the bloodstream is bound to sex hormone-binding globulin (SHBG). Free testosterone, on the other hand, accounts for approximately 2% of the total cholesterol in circulation and is taken up by tissues to bind with androgen receptors (ARs) and mediate regenerative processes [67].

The nature of the impact of physical activity on the male hormonal system is complex. Indeed, some studies do not show the existence of a relationship between sporting activity and hormone production [68,69], while others show either a negative [70] or a positive [71] association. A study performed on a sample of the US population indicates a reduced risk of low testosterone levels in males performing light to moderate physical activity [72]. However, this is inconsistent with the findings of a comprehensive study performed by Safarnejad et al. [73], in which two training protocols were used, i.e., a moderate-intensity (60% VO2 max) and a high-intensity (80% VO2 max) protocol. The study showed that the levels of LH, FSH, and testosterone, including free testosterone, significantly decreased after 12 weeks in both training groups. This reduction in hormone levels could be connected with the exercise-induced suppression of serum testosterone, which is associated with suppressed stimulation of gonadotropin release caused by gonadotropin-releasing hormone (GnRH) during exercise, as well as reduced testicular ability to secrete testosterone during the recovery period [74]. However, Safarnejad et al. [73] also claimed that the levels of LH, FSH, and testosterone could be restored after 60 weeks of moderate- to high-intensity training, followed by 36 weeks of low-intensity training, treated as the recovery period. On the contrary, the results of a study performed by Vingren et al. [75] showed increased serum testosterone levels in subjects who performed moderate-intensity physical exercises. Another method for maintaining testosterone levels proposed in the literature is magnesium supplementation (10 mg/kg body weight), which improves its levels in both exercising and sedentary males [76]. As far as particular exercise modes are concerned, it has been shown that free-weight lifting has a more beneficial influence on testosterone levels compared to training on machines [77]. Increased testosterone levels were also found in adolescent boys subjected to 30 min. of moderate physical activity compared to the control group, who were involved in the same amount of sedentary behavior. The latter mode of activity has also been found to increase cortisol levels in the subjects [78]. In contrast, Steeves et al. [79] found no association between physical activity and testosterone levels but noted that although increased physical activity was not found to be linked to testosterone levels, it may be associated with a lower likelihood of low or low–normal testosterone in non-obese men, a relationship not observed in obese men. The long-term effects of physical activity, however, are less well understood, with some evidence suggesting lower testosterone levels in endurance athletes [80].

Other studies have attempted to determine the relationship between physical exercise and the HPG axis [25,81]. Vaamonde et al. [25] demonstrated that under controlled conditions, no disruption of the hormonal balance involving the HPG axis occurs; on the contrary, an improvement in semen quality in physically active individuals was observed. The HPG axis plays a crucial role in the body’s response to exercise and training—both in the short and in the long term—and has an impact on testosterone levels in men. A crucial factor that influences the proper functioning of the HPG axis is sex hormone-binding globulin. However, studies focusing on the impact of physical activity on SHBG do not reach a consensus regarding changes in the concentrations of HPG under training conditions, Safarinejad et al. [73] reported a significant increase in SHBG, both in the case of high- and moderate-intensity training. Moreover, higher SHBG levels were associated with lower levels of free testosterone in the serum of the subjects. In another study, men characterized by high levels of physical activity had 14% and 8% (6% and 3% after adjusting for BMI) higher concentrations of SHBG and total testosterone, respectively, compared to men whose physical activity was lower [82]. The cause of the aforementioned endocrinological dysfunctions following intense physical exercise appears to be the production of reactive oxygen species in the body of the individual undergoing training [83,84], which links endocrine effects of physical exercise with those involving the immune response.

It is worth emphasizing that thyroid disorders have also been shown to have an adverse impact on fertility. Bahreiny et al. [85] indicated that subclinical hyperthyroidism (SCH), i.e., a mild thyroid disorder characterized by low serum thyroid-stimulating hormone (TSH) levels while normal thyroid hormone levels remain within the normal range, has a negative influence on the levels of reproductive hormones and semen quality, with decreased LH levels, elevated FSH levels, and decreased TSH levels, among others. Moreover, hypothyroidism can affect the secretion of GnRH through increased prolactin levels, leading to hypogonadotropic hypogonadism [86]. Wu et al. [87] reported that exercising is closely linked to maintaining thyroid homeostasis in patients with subclinical hypothyroidism. It should also be noted that men experiencing hypothyroidism can also display reduced libido or erectile dysfunction [88,89]. For example, Jaya Kumar et al. [88] found a prevalence of libido reduction in 37.5% of hypothyroid patients, while Carani et al. [89] reported low sexual desire, impaired erectile function, and delayed ejaculation in hypothyroid men. In addition, the literature data show that while thyroid function may be impaired by excessive physical activity [90], low-level activity, such as increasing the daily number of steps, can reduce the risk of thyroid hormonal imbalances [91]. These findings corroborate the notion that physical activity should be tailored independently to the needs of each individual when thyroid disorders are concerned.

### 3.3. Impact of Physical Activity on Fertility Through Its Effect on the Circulatory System

Cardiovascular diseases (CVDs) are a major global health concern, with risk factors such as dyslipidemia, hypertension, and diabetes potentially affecting male fertility [92]. Uncontrolled dyslipidemia and the associated inflammation can impair endothelial function and hormone regulation, both of which are crucial for reproductive health [29]. In contrast, improving cardiovascular fitness through exercise may help reduce these risks and thus have a positive impact on fertility [92].

One of the most widely recognized associations between physical activity, cardiovascular health, and male fertility is through problems with erection. The underlying hypothesis is that physical activity can improve blood flow through the testes by inhibiting atherosclerosis, boosting vascular blood delivery, refining the lipid profile, and reducing the risk of thrombosis [93]. In this context, erectile dysfunction, indeed, often serves as a warning sign for underlying cardiovascular problems. For this reason, assessing patients’ individual cardiovascular risk is essential before physical exercise is considered as a viable remedial measure for problems with erection [94], as it may potentially lead to the exacerbation of cardiovascular issues. However, individuals whose problems with erection have been reliably linked with impaired cardiovascular health through proper medical assessment may benefit from performing physical activity [95]. This may enhance both their reproductive as well as cardiovascular health.

Physical exercise is generally regarded as a fundamental component of a healthy lifestyle and, outside the scope of reproductive medicine, this role is often linked to cardiovascular effects such as the reduced risk of stroke or myocardial infarction. Thus, although physical activity is recognized to have beneficial effects on health, including its cardiovascular aspects, it is important to note that intense or excessive exercise can be detrimental, which also concerns the area of male fertility [2,26,28]. However, studies do not link this negative influence directly with cardiovascular effects, as immune and endocrine processes are those that are typically indicated as key in the context of the negative association between excessive physical activity and male fertility [29,96]. Studies show that a balanced approach to physical activity—moderate and consistent—improves cardiovascular function and reduces the risk of chronic diseases, thus having a potentially beneficial influence on male fertility.

Overall, the impact of physical activity on male fertility through its association with cardiovascular health appears to be less pronounced compared to other processes. The main association is through the enhanced blood flow to the reproductive organs resulting from performing physical exercises. Hence, regular exercise helps improve circulation, maintain healthy blood vessels, and regulate blood pressure, all of which are crucial for the proper functioning of the body, including the testes and other reproductive structures. In addition, improved circulation supports the delivery of oxygen and nutrients to the testes, which is essential for optimal spermatogenesis. Physical activity can also help reduce the risk of conditions associated with impaired circulation and impaired fertility, such as obesity and diabetes.

### 3.4. Impact of Physical Activity on Fertility Through Its Action Related to Insulin Resistance, Diabetes, and Obesity

Numerous studies point to complex negative associations between diseases and conditions connected with glucose metabolism, i.e., diabetes, insulin resistance, and obesity, and fertility [38,97]. Due to the fact that metabolic conditions are exacerbated by a lack of exercise, it can be safely assumed that increased physical activity may have a beneficial effect on male fertility, e.g., by improving glucose metabolism. The literature data show that exercise can enhance male fertility in conditions of lifestyle-induced infertility, such as obesity and diabetes [2]. Hence, adopting a healthy lifestyle that encompasses balanced nutrition, physical activity, and effective weight management is the key strategy for managing metabolic disorders and promoting optimal male fertility [37]. It should be noted, however, that the precise interactions between these three aspects are not yet sufficiently studied, with the need for robust studies such as randomized controlled trials (RCTs) focused on the male population [97].

It is commonly agreed that the impact on insulin resistance, diabetes, and obesity on male fertility is negative. A systematic review performed by Service et al. [28], who analyzed 112 articles published between 2013 and 2023, indicates that obesity, diabetes, and metabolic syndrome adversely affect multiple aspects of male fertility, including semen quality and sperm DNA integrity. Specifically, research points to the association between insulin resistance and sperm motility [98]. Additionally, growing evidence suggests the existence of a negative association between male obesity and live birth rates, whether through natural conception or assisted reproductive techniques (ARTs). Moreover, obesity has been shown to disturb the balance between oxidation and antioxidation in the testes, leading to oxidative stress, which activates NF-κB, initiating an inflammatory response. As mentioned earlier, this leads to a decrease in testosterone production and the worsening of sperm quality [3].

As mentioned above, obesity is largely agreed to have a detrimental impact on male fertility [23]. Most importantly, increased ROS production is commonly observed in obese men, in whom low levels of circulating testosterone can also be routinely observed. Kumagai et al. [99] showed that a 12-week aerobic exercise program raised serum levels of total testosterone, free testosterone, and bioavailable testosterone in overweight or obese men, concluding that vigorous physical activity boosted circulating testosterone levels in this group. It has been well documented that aerobic physical activity reduces insulin resistance associated with hypogonadism and improves erectile function [100,101,102,103]. Based on the principle that exercise-induced weight loss has a positive impact on reproductive hormones by regulating metabolic pathways, Lo Giudice et al. [18] performed a meta-analysis of RCTs, whose results show that physical activity is associated both with improved semen parameters and fertility outcomes.

It is interesting to note that despite the rather well-established finding that excessive exercise is detrimental to fertility, this may not be in fact true for obese males. Studies show that endurance exercises and high-intensity interval training have a positive influence on glucose metabolism in obese males [3]. In addition, a large study performed by Fantus et al. [104] in a group of 7372 men found that those who exercised more intensely than the recommended level of activity had a lower risk of low testosterone levels than those who did not meet the recommended levels of exercise. Although this finding may be pointing to a beneficial impact of excessive physical activity on obese subjects, it must be also remembered that obese persons are mostly unable to perform extremely strenuous exercises, e.g., due to an increased risk of cardiovascular events, and thus, what is considered excessive for them may be moderate for men who are fitter.

### 3.5. Impact of Physical Activity on Fertility Issues Associated with Its Physical Action on the Male Body

Certain types of activities may negatively affect male fertility due to overheating [105], insufficient oxygen levels during physical exercise [106,107], or pressure and trauma [108]. In terms of adequate temperature levels, the ideal temperature for spermatogenesis is approximately 2 °C below the core body temperature [109]. Studies have shown that elevated scrotal temperature may increase the production of ROS, which can damage the sperm plasma membrane and lead to DNA fragmentation [110]. In addition, animal studies suggest that an increase in testicular temperature by 1–1.5 °C leads to a reduction in sperm production, testicular shrinkage, a predisposition to the production of morphologically abnormal sperm, and a lower concentration of sperm with normal motility [111,112]. Figà-Talamanca et al. [113] found that two hours spent in a sedentary posture leads to an increase in testicular temperature of approximately 2 °C, which suggests that prolonged time spent, for instance, in a car may increase the risk of reproductive problems. Excessive use of hot baths or saunas has also been shown to have an adverse impact on male fertility [114]. It should be noted, however, that some studies do not corroborate the link between the increased temperature resulting from sedentary lifestyle and a deterioration in semen parameters [27]. It may also be interesting to mention that research conducted on rats indicates than males are more susceptible to the effects of exercise-induced overheating than females [109], which may point to a biological mechanism that makes overly strenuous training incompatible with male fertility specifically.

Interestingly, the literature data point to the fact that exposure to excessively high temperatures may have a detrimental effect on male fertility in a similar manner to the effects of physiological aging [22]. Human sperm is produced even at an advanced age, but when the decision is made to start a family later in life, certain changes must be taken into account. A decline in semen quality among older fathers is already observed from the age of 40, with a reduction in the number of sperm with normal morphology [115], After the age of 45, a gradual decline in semen volume is also observed, likely due to a functional impairment of the accessory glands [116]. For this reason, it can be stated that older men may be characterized by impaired testicular function [117], a decreased production of sex hormones [118], and reduced semen quality parameters [22,119]. These changes may also contribute both to a higher incidence of congenital abnormalities in fetuses and their mortality [106,107]. The results of a study by Johnson et al. [105] show that men over the age of 75 had an average testicular volume 31% smaller compared to men aged 18 to 40 years. Other studies indicate that the average total number of Leydig cells decreased by as much as half in men aged 50–76 compared to men aged 20–48 [108].

In this context, regular physical activity, when practiced over the long term, may have a protective effect against the oxidative stress associated with aging [120,121]. Zhao et al. [120] demonstrated that physical exercise leads to better regulation of the mRNA levels of the enzymes SOD, CAT, and GPx. In addition, mice subjected to exercise as part of the research exhibited improved regulation of testosterone levels. In the study by Kraemer et al. [122], after a 10-week strength–power training program, an increase in free and total testosterone levels was observed in participants around 60 years of age, which was not seen in individuals around 30 years old following the same training program. On the other hand, Häkkinen et al. [123] observed no changes in testosterone secretion after training in either older or younger groups. Although both results were contrasting, this could be explained by the fact that different training regimes were used, with one being weightlifting and the other heavy resistance training. Exercise in resistance training involves pushing or pulling against the resistance of an object (including one’s own body), while strength training involves increasing the amount of muscle tissue by continuously increasing the lifted weight, while decreasing the number of repetitions. The latter type of exercise leads to bigger body gains in strength, which may, in turn, result in differences in testosterone production.

In addition to temperature, other factors can have a detrimental effect on male fertility, including inadequate oxygen intake. High-altitude sports, among others, are associated with the risk of hypoxia [29,124], which is linked to testicular dysfunction [29], as is deep diving. In the study by Aitken et al. [125], sperm motility and sperm concentration deteriorated shortly after a diving episode and remained impaired for up to 3 months.

Performing sports is also connected with increased pressure, and potentially even trauma, to the gonadal region. For example, cycling can negatively affect male fertility through both pressure and increased temperature. Furthermore, regular use of tight-fitting clothes, such as racing suits, during cycling and the pressure exerted on the scrotum by the saddle adversely impact the fertility of cyclists. Additionally, the increased risk of pelvic floor injuries in cycling further contributes to the negative effects of this particular sport on fertility by influencing the blood flow in the dorsal vein complex. Worsened blood flow in that area can lead to a weakened capillary washout of prostate-specific antigen (PSA) into the circulation [126,127]. It should also be noted that wearing tight underwear may have a similar impact on fertility to those described above [128].

Sgrò and Di Luigi [95] make a valid point by indicating the numerous ways in which competitive sports can cause damage and dysfunction to the reproductive and sexual tracts. These negative impacts can be either temporary—i.e., presenting as genital pain, reduced sensation, hypogonadism, erectile dysfunction, changes in sexual drive, among others, or permanent. All of them can occur through direct factors, such as trauma to the external genitalia—as in the case of the aforementioned saddle-related issues in cyclists—or indirect factors, including exercise-induced hypogonadism, substance abuse, doping, and stress, among others.

As far as the effects of physical activity on the male body are concerned, it can be concluded that to ensure its positive impact on male fertility, the choice of sports disciplines must be carefully considered. As certain incidental factors, such as wearing tight-fitting clothes, excessive pressure, or oxygen deprivation, may have a detrimental effect on fertility, a hypothesis worth exploring would be whether the positive effects of physical exercise may, to some extent, be counterbalanced by the aforementioned secondary aspects of intense physical activity. As such factors are particularly common in professional sports, they may contribute to the negative effects of excessive exercise on male fertility reported in the scientific literature.

## 4. Conclusions

Physical activity plays a multifaceted role in supporting male fertility by positively influencing the body’s key physiological systems. Moderate and regular exercise enhances cardiovascular health, improving blood flow and oxygen delivery to the reproductive organs, which are essential for optimal spermatogenesis and hormone function. In addition, exercise supports hormonal balance by modulating the hypothalamic–pituitary–gonadal axis and regulating the levels of key hormones, including testosterone, luteinizing hormone (LH), and follicle-stimulating hormone (FSH). Physical activity also strengthens immune function, reduces systemic inflammation, and improves antioxidant defenses, thus protecting reproductive tissues from oxidative stress and damage.

Apart from its direct benefits, physical activity mitigates lifestyle-related factors contributing to infertility, such as obesity, insulin resistance, and metabolic syndrome, by improving glucose metabolism and supporting healthy weight management. However, excessive or overly intense exercise can disrupt hormonal equilibrium and induce oxidative stress, negatively affecting semen quality. Temperature, pressure, and problems with oxygen—factors most often associated with intense training and professional performance—may further contribute to the negative effects of high-level exercise. For this reason, studies focused on establishing the extent to which the negative effects of intense physical activity are connected with incidental factors connected with professional sports, e.g., tight-fitting clothes, could be a viable research direction.

A balanced approach to exercise is critical for maximizing its benefits while minimizing potential risks, making physical activity a vital component of strategies aimed at enhancing male reproductive health, especially in the case of obese males. Based on the available literature data, it is safe to conclude that recreational and well-designed exercise regimes could have a beneficial influence on male fertility, while the impact of excessive training programs and competitive sports is more likely detrimental.

## 5. Limitations of the Study

This study has several limitations, most of which stem from the nature of its subject. First of all, the impact of physical exercise on male fertility is rarely studied in rigorous clinical settings, with observational studies being more common. Hence, confounders such as the intensity of the participants’ physical activity prior to the study or their activity during the development of their reproductive systems cannot be accounted for, as well as other lifestyle-related conditions and choices concerning, e.g., diet or environmental conditions. As a result, the findings are often uncertain, and many studies remain preliminary. Additionally, infertility is a multifaceted issue, involving complex biological, biochemical, and methodological factors, which means that most studies do not provide conclusive evidence to establish clear associations. Moreover, no clear consensus of what constitutes ‘excessive’ physical activity exists throughout the reviewed articles; similarly, the training regimes used in different studies may differ despite the fact that they are described using similar terms.

## Figures and Tables

**Table 1:** The impact of various aspects of the human biology on male fertility.

Aspect	Mechanism Associated with the Impact on Male Fertility	Positive Influence on Male Fertility	Negative Influence on Male Fertility	Main Conclusion
Immunological	Physical activity supports immune function by reducing inflammation and improving antioxidant defenses, which can protect reproductive tissues from oxidative stress and enhance semen quality	Regular exercise enhances semen quality, reduces oxidative stress, and promotes spermatogenesis by improving testicular antioxidant defenses and modulating immune response	Excessive exercise can cause an imbalance between oxidative stress and antioxidant defense, leading to potential sperm damage, DNA fragmentation, and reduced fertility; a lack of physical activity leads to worsened antioxidant defense, leading to sperm DNA fragmentation	Balanced exercise positively affects fertility by supporting immune function and reducing inflammation; excessive activity may cause oxidative damage and negatively affect fertility
Endocrine	Exercise modulates the hypothalamic–pituitary–gonadal (HPG) axis, influencing hormone production (e.g., testosterone, LH, and FSH) crucial for sperm production and sexual function	Moderate physical activity supports hormonal balance, stimulating spermatogenesis and improving testosterone levels	Intense or prolonged physical activity can suppress the HPG axis, reducing testosterone, LH, and FSH levels, which can impair fertility; sedentary behavior may lead to lower testosterone levels	Regular, moderate exercise improves hormonal regulation and fertility; excessive exercise can disrupt hormonal balance and negatively impact reproductive health
Circulatory	Physical activity improves cardiovascular health, which is crucial for erectile function and blood flow to the reproductive organs; inadequate circulation can lead to erectile dysfunction, which is a common indicator of cardiovascular issues	Regular exercise enhances cardiovascular health, improving blood flow to the testes and erectile function, which supports overall fertility	Excessive or intense exercise can exacerbate existing cardiovascular issues, potentially worsening erectile dysfunction and, indirectly, male fertility; a lack of physical activity may lead to the development of metabolic syndrome and worsened erectile function	Regular, moderate exercise is beneficial for erectile function and fertility by improving cardiovascular health and blood circulation, while excessive exercise may negatively impact erectile health and fertility through cardiovascular complications
Insulin resistance, diabetes, and obesity	Physical activity improves insulin sensitivity, reduces the risk of diabetes and obesity, and enhances metabolic health, which are critical factors in male fertility	Regular physical activity reduces insulin resistance and helps maintain proper body weight, which can improve sperm quality and hormone levels, particularly in individuals with obesity or metabolic disorders	While no direct negative impacts of exercise on fertility in diabetic or obese males were found, extreme exercise can exacerbate metabolic disorders or lead to fatigue and oxidative stress; the level of physical activity should be carefully considered in the case of obese males; a lack of physical activity leads to the development of obesity, insulin resistance, and diabetes, which can impair overall fertility	Physical activity has a positive impact on fertility, especially by managing metabolic conditions such as insulin resistance, obesity, and diabetes, which negatively affect reproductive health
Physical action	Intense, especially professional, physical activity may lead to physical stress on the body, such as overheating, insufficient oxygen levels, and trauma to the reproductive organs, which can disrupt sperm production and testicular function	Moderate physical activity can improve general health and reproductive function through better tissue repair, improved hormone levels, and overall well-being	Intense physical activity can cause overheating (e.g., from excessive heat in cycling or saunas) in a manner similar to aging, oxygen deprivation (e.g., from high-altitude sports), or trauma (e.g., from pressure in cycling or sports injuries), all of which negatively impact sperm production and testicular function	Regular, moderate physical activity is beneficial for fertility, while excessive physical activity may have detrimental effects, particularly through factors connected with professional sports
